# HIV-Infected Children Living in Central Africa Have Low Persistence of Antibodies to Vaccines Used in the Expanded Program on Immunization

**DOI:** 10.1371/journal.pone.0001260

**Published:** 2007-12-05

**Authors:** Mathurin C. Tejiokem, Ionela Gouandjika, Lydie Béniguel, Marie-Claire Endegue Zanga, Gilbert Tene, Jean C. Gody, Elisabeth Njamkepo, Anfumbom Kfutwah, Ida Penda, Catherine Bilong, Dominique Rousset, Régis Pouillot, Frédéric Tangy, Laurence Baril

**Affiliations:** 1 Centre Pasteur du Cameroun, Laboratoire d'Epidémiologie et de Santé Publique, Yaoundé, Cameroun; 2 Centre Pasteur du Cameroun, Laboratoire de Virologie, Yaoundé, Cameroun; 3 Centre Pasteur du Cameroun, Laboratoire d'Analyses Médicales, Yaoundé, Cameroun; 4 Institut Pasteur de Bangui, Laboratoire des Entérovirus, Bangui, Central African Republic; 5 Institut Pasteur de Bangui, Laboratoire des Rétrovirus, Bangui, Central African Republic; 6 Centre Mère et Enfant de la Fondation Chantal Biya, Yaoundé, Cameroun; 7 Complexe Pédiatrique de Bangui, Central African Republic; 8 Centre National de Référence des Bordetella, Institut Pasteur, Paris, France; 9 Laboratoire de Génomique Virale et Vaccination, Institut Pasteur, Paris, France; 10 Unité d'Epidémiologie des Maladies Emergentes, Institut Pasteur, Paris, France; 11 Hôpital Laquintinie, Service de Pédiatrie, Douala, Cameroun; University of Cape Town, South Africa

## Abstract

**Background:**

The Expanded Program on Immunization (EPI) is the most cost-effective measures to control vaccine-preventable diseases. Currently, the EPI schedule is similar for HIV-infected children; the introduction of antiretroviral therapy (ART) should considerably prolong their life expectancy.

**Methods and Principal Findings:**

To evaluate the persistence of antibodies to the EPI vaccines in HIV-infected and HIV-exposed uninfected children who previously received these vaccines in routine clinical practice, we conducted a cross-sectional study of children, aged 18 to 36 months, born to HIV-infected mothers and living in Central Africa. We tested blood samples for antibodies to the combined diphtheria, tetanus, and whole-cell pertussis (DTwP), the measles and the oral polio (OPV) vaccines. We enrolled 51 HIV-infected children of whom 33 were receiving ART, and 78 HIV-uninfected children born to HIV-infected women. A lower proportion of HIV-infected children than uninfected children had antibodies to the tested antigens with the exception of the OPV types 1 and 2. This difference was substantial for the measles vaccine (20% of the HIV-infected children and 56% of the HIV-exposed uninfected children, p<0.0001). We observed a high risk of low antibody levels for all EPI vaccines, except OPV types 1 and 2, in HIV-infected children with severe immunodeficiency (CD4^+^ T cells <25%).

**Conclusions and Significance:**

Children were examined at a time when their antibody concentrations to EPI vaccines would have still not undergone significant decay. However, we showed that the antibody concentrations were lowered in HIV-infected children. Moreover, antibody concentration after a single dose of the measles vaccine was substantially lower than expected, particularly low in HIV-infected children with low CD4^+^ T cell counts. This study supports the need for a second dose of the measles vaccine and for a booster dose of the DTwP and OPV vaccines to maintain the antibody concentrations in HIV-infected and HIV-exposed uninfected children.

## Introduction

Pediatric HIV infection is a major public health threat. Two thirds of the 700,000 [630,000 to 820,000] children less than 15 years old newly infected with HIV in 2005 were living in sub-Saharan Africa [1]. Mother to child transmission of HIV is still a major route of infection for children. This is related mainly to insufficient access to prevention methods, HIV screening and antiretroviral treatment (ART) in developing countries. Without appropriate ART, HIV-infected children experience progressive immune depression and become susceptible to infectious diseases, some of which could be prevented by immunization.

The World Health Organization (WHO) recommendations for immunization of HIV-infected children differ slightly from the general guidelines for HIV-uninfected children [2]. The use of vaccines in HIV-infected and HIV-exposed uninfected children raises questions about the capacity of those children to mount and maintain efficacious antibody levels. Several clinical trials in HIV-infected children report low antibody levels to various vaccines [reviewed in 3], [4].

In this study, we evaluated the persistence of antibody levels in HIV-infected and HIV-exposed uninfected children born to HIV-infected mothers, living in Central Africa and who previously received EPI vaccines in routine clinical practice. In addition, we assessed the influence of host and viral related factors (nutritional, clinical and biological) on the association between HIV infection and the absence of antibody to EPI vaccines.

## Methods

### Participants

We conducted a cross-sectional study in 4 pediatric care centers (3 in Cameroon and 1 in the Central African Republic (CAR)). Children were recruited from November 2004 to June 2005. Children were eligible if: (i) they were between 18 and 36 months of age; (ii) they were born (not prematurely, *i.e*. ≥ 37 weeks) to an HIV-infected mother who was diagnosed in the context of the mother to child transmission prevention program; and (iii) they had received at least a primary vaccine series of three doses of the combined diphtheria, tetanus, and whole-cell pertussis (DTwP) vaccine, the total oral polio (TOPV) before one year of age, and one dose of the measles vaccine (documented on an immunization card). The HIV-1 status has been already confirmed by serology in all included children between 15 and 18 months of age. Children were not included in the study if (i) their clinical status was severely impaired (including advanced or severe HIV-associated symptoms) or (ii) they were breastfed six months before the enrolment period to avoid recent HIV infection through breastfeeding.

The EPI schedule in both countries included BCG vaccination and one dose of TOPV (dose 0) given at birth; a primary vaccination with three doses of the DTwP combined vaccine and TOPV given at 6, 10, and 14 weeks; and recommended booster doses of the DTwP combined vaccine and TOPV given at 18 months of age. The measles vaccine was scheduled to be given at nine months of age and a second dose was recommended at 12 to 15 months of age.

### Ethics

All parents or legal guardians gave written informed consent. The study was approved by National Ethics Committees in Cameroon and in CAR. The study was conducted in accordance with the Declaration of Helsinki.

### Laboratory techniques

Blood samples were collected and processed at the Centre Pasteur in Yaoundé (Cameroon) and at the Institut Pasteur in Bangui (CAR). Complete blood count, proteinemia and tests for HIV-1-infected children including assessment of HIV-1 viral load and lymphocyte subpopulation counts were done. Thick blood smears were also performed for malaria diagnostics. In Cameroon, the plasma HIV-1 RNA viral load (VL) was quantified by a commercial assay (Versant® bDNA HIV kit version 3.0, Bayer Diagnostics, Emeryville, CA, USA) according to the manufacturer's instructions. The threshold for quantification was 50 HIV-1 RNA copies/ml. In Bangui, values for the plasma HIV-1 RNA were determined by real-time TaqMan RT-PCR with the protocol established by the Working Group for Viral Quantification of the ANRS [5]. The limit for quantification was 400 HIV-1 RNA copies/ml. The CD4^+^ T cells were counted with a fluorescence activated cell sorter (FacScan) flow cytometer (Becton Dickinson Biosciences, San Jose, CA, USA).

The antibody concentrations or titers to measles, diphtheria, tetanus, and polio types 1, 2 and 3 were analyzed at the laboratory sites. Serological testing for pertussis was performed by the French National Reference Center for *Bordetella*. All the samples were tested in duplicates, and results were expressed as the mean values for the two determinations. The antibody level was considered adequate if the antibody concentration or titer was above the cut-off value defined by the manufacturers or by WHO recommendations.

Antibodies to measles were initially measured with a commercial ELISA kit (Enzygnost Anti-measles virus/IgG, Dade Behring, Germany) in which plates were coated with inactivated measles virus [6]. The assay was calibrated using an international reference reagent for the measles vaccine (National Institute for Biological Standards and Control (NIBSC) #92/648, Hertfordshire, UK). The results were classified as negative (ΔOD <100 mUI/ml) or equivocal (100 ≤ ΔOD ≤ 335 mUI/ml). Quantitative values were calculated for positive samples (ΔOD>335 mUI/ml). Because the percentage of positive samples was low in both groups (16% and 62.3% of in HIV-infected and uninfected children, respectively) with this initial technique, all samples were re-tested for antibodies to measles using an IgG capture enzyme immunoassay (Measles IgG capture EIA, Microimmune Ldt, Brentford, UK) [7]. This qualitative assay was done by the Laboratory of Virology at the University Hospital of Caen (France), the French Associated Reference Center for Measles. Briefly, plates were coated with anti-human IgG and a recombinant measles nucleoprotein antigen was used to detect specific IgG against measles. Sera were identified as IgG positive if the OD was at least 1.25 times higher than the mean OD of three negative control wells.

Antibodies to diphtheria and tetanus toxoids were measured using commercial ELISA kits (Diphteria ELISA IgG Testkit and Tetanus ELISA IgG Testkit, Genzyme Virotech, Russelsheim, Germany). Sera with antibody concentrations of, at least, 100 mIU/ml were considered positive. For the wP vaccine, agglutinins (AGG), i.e. antibodies that agglutinate bacteria mostly directed against fimbrial antigens, were measured. These AGG were detected by the classical micro-agglutination test [8]. Adequate results were defined as values equal to or superior to the value for the first dilution tested (1/20 dilution).

Neutralizing antibody titers against each of the three poliovirus types were measured using a micro-neutralizing assay, based on the WHO recommended procedure [9]. The potency of the in-house reference serum (identical for both sites) was assayed in parallel with the second International Reference Reagent for live attenuated poliovirus (NIBSC #66/202) so that the results could be converted to International Units (IU). Two-fold dilutions of sera ranging from 1/8 to 1/1024 were incubated for three hours at 36°C in triplicate with 100 median TCID_50_ doses of the corresponding Sabin-type poliovirus. HEp-2 cells were added and plates were scored after five days. The neutralizing antibody titer was the end point dilution of sera that protected 50% of the cell culture. Detectable antibody at a dilution of 1∶8 was considered positive for that poliovirus serotype (equivalent to 0.6 IU for poliovirus 1, 1.1 IU for poliovirus 2 and 0.3 IU for poliovirus 3). The geometric mean concentration (GMC) for each poliovirus serotype was calculated as IU for all samples.

### Statistical methods

The chi-square or Fisher's exact test was used as appropriate to compare categorical variables of HIV-infected and uninfected children. The student's t-test was used to compare continuous variables if the data adhered to normality assumptions; otherwise the Mann-Whitney test was used. The results for antibodies to the DTwP vaccine, the measles vaccine and the TOPV were reported as the GMC or GM titers (GMT) with the 95% confidence intervals (CI) for each group.

Univariate and multivariate logistic regression models were used to assess the effect of covariates on the odds ratios (OR) of a low antibody concentration to vaccine (only the serotype 3 for the OPV). Composite categorical variables were created to evaluate the effect of HIV infection: i) HIV status combined with the percentage of CD4^+^ T cells (HIV-exposed uninfected, HIV infected and ≥25% CD4^+^ T cells, HIV infected and <25% CD4^+^ T cells) [10]; ii) HIV status combined with HIV VL (HIV-exposed uninfected, HIV infected and VL <10,000 copies/ml, HIV infected and VL ≥10,000 copies/ml) [11]; and iii) HIV status combined with duration of ART (HIV-exposed uninfected, HIV infected and ≥6 months ART, HIV infected and <6 months of ART or no ART). Six months was chosen as a reasonable ART period to declare failure and to, eventually, start a second line of ART in resource–constrained countries. The perinatally HIV-exposed uninfected children constituted the reference group in this study. STATA version 8.0 (Stata Corp, College Station, TX, USA) was used for all statistical analyses, with a significance level of 5%.

## Results

### Study population

The first two criteria for eligibility were met by 177 children (54 HIV-infected and 123 HIV-exposed uninfected), but the third criteria (EPI schedule) was not fulfilled in three HIV-infected and 45 HIV-exposed uninfected children (44 from CAR). Thus, 129 children were included in the study: 51 perinatally HIV-infected (16 in the CAR and 35 in Cameroon) and 78 HIV-exposed uninfected (44 in the CAR and 34 in Cameroon). The mean age of children enrolled was 24.6 months (90% range; 18.1 to 35.6). Sex ratio, duration of breastfeeding, weight, MUAC [9], number of vaccine doses, and the frequency of positive blood smears for malaria did not statistically differ between the two groups. The median time interval between the collection of blood for antibody determination and the last vaccination was 17.3 months (90% range; 3.9 to 31.3 months) for the DTwP vaccine and 12.8 months (90% range; 3.3 to 26.1 months) for the measles vaccine.

According to the WHO pediatric clinical staging system [12], 36 (70%) of the HIV-infected children were classified as stage 1 and 15 (29.9%) at stage 2. Almost two thirds of them (33/51) were receiving ART at the time of inclusion in the study (six in CAR and 27 in Cameroon); the duration of treatment was at least six months for 61% (20/33) of the children receiving ART. The mean age of children starting ART was 18.1 months (90% range: 7.3–34.2 months), indicating that the majority of the children began ART several months after they received the first series of the EPI vaccines. The most frequent ART combinations were AZT+3TC+NVP (n = 22) and D4T+3TC+NVP (n = 8). In HIV-infected children, 61% (31/51) had a plasma VL below 10,000 copies per ml (6 in the CAR and 25 in Cameroon). For 51 HIV-infected children, the distribution according to the percentages of CD4^+^ T cells was as follows: <15% (n = 15), 15–24% (n = 16) and ≥ 25% (n = 20).

### HIV-infected children have low levels of antibodies to EPI vaccines

We observed that the proportion of HIV-infected children with adequate antibody levels to all the tested vaccine antigens was lower than that of HIV-exposed uninfected children (Table 1). The difference was statistically significant for all tested antigens with the exception of poliovirus types 1 and 2. The GMCs were lower in the HIV-infected group than in the HIV-exposed uninfected group for all tested vaccines with the exception of the measles vaccine. These differences were statistically significant for tetanus and poliovirus types 1 to 3. Few children received a booster of the DTwP vaccine (15%) or a second dose of the measles vaccine (7%). Only 20% (three HIV-infected and 21 HIV-exposed uninfected) of these children had the expected antibody levels against all the tested vaccines, 71% (37 HIV-infected and 55 HIV-exposed uninfected) had low antibody levels to one or more vaccine antigens, and 9% (9 HIV-infected and 1 HIV-exposed uninfected) did not develop or maintain the expected antibody level to the vaccine antigens. Moreover, the proportion of children with low antibody levels to EPI vaccines differed according to the degree of immunodeficiency, except for the tetanus and the poliovirus type 3 vaccines (Table 2). Children with CD4^+^ T-cell counts lower than 25% had significantly low levels of antibodies, particularly to the live attenuated measles vaccine. A short duration of ART or no ART was significantly associated with a low measles antibody concentration (tested by EIA) as shown in the Table 3. Severe immunodeficiency (<25% CD4^+^ T cells, HIV VL ≥10,000 copies) was also associated with a low antibody concentration. Only the covariates linked to HIV status (ART, % CD4^+^ T cells, HIV VL, macrocytosis, protidemia) were statistically significant. More children with low measles antibody concentration than those with adequate antibody concentrations were breastfed; however, the difference was weakly significant.

**Table 1:** Proportions of adequate antibody concentrations or titers to the EPI vaccines and geometric mean concentrations or titers for antibodies against measles, DTwP and TOPV antigens, according to the HIV status of the children.

Vaccine antigens		HIV-infected children			HIV-exposed uninfected children			*P*1*	*P*2†
	Number of doses	N	Adequate responses n (%)	GMC ‡ [95% CI]	N	Adequate responses n (%)	GMC‡ [95% CI]		
Measles									
*Enzygnost*	Total	50	8 (16.0)	1091.5 [576.1–2067.8]	77	48 (62.3)	943.2 [782.7–1136.6]	<10^−4^	0.56
	1 dose	46	7 (15.2)		72	45 (62.5)		<10^−4^	
	2 doses	4	1 (25.0)		5	3 (60.0)		0.36	
*Microimmune*	Total	49	10 (20.4)		77	43 (55.8)		<10^−4^	
	1 dose	45	9 (20.0)		72	39 (54.2)		<10^−4^	
	2 doses	4	1 (25.0)		5	4 (80.0)		0.21	
Diphtheria	Total	51	27 (52.9)	245 [166–361]	78	62 (79.5)	312 [246–396]	0.001	0.13
	3 doses	44	22 (50.0)		66	51 (77.3)		0.003	
	4 doses	7	5 (71.4)		12	11 (91.7)		0.29	
Tetanus	Total	51	43 (84.3)	289 [228–367]	78	76 (97.4)	491 [396–609]	0.01	0.001
	3 doses	44	36 (81.8)		66	64 (97.0)		0.01	
	4 doses	7	7 (100.0)		12	12 (100.0)			
Pertussis	Total	50	15 (30.0)	52.8 [41.4–67.3]	78	43 (55.1)	62.8 [52.2–75.6]	0.005	0.28
	3 doses	43	10 (23.3)		66	34 (51.5)		0.003	
	4 doses	7	5 (71.4)		12	9 (75.0)		0.63	
TOPV	≥4 doses								
Serotype 1		51	48 (94.1)	6.1 [4.5–8.3]	78	78 (100.0)	16.1 [12.1–21.5]	0.06	<10^−5^
Serotype 2		51	48 (94.1)	13.2 [ 8.9–19.3]	78	77 (98.7)	42.5 [32.5–58]	0.17	<10^−5^
Serotype 3		51	36 (70.6)	2.3 [1.3–3.9]	78	75 (96.2)	4.5 [3.3–6.1]	<10^−4^	0.02

* *P*1: P value for the comparison of the proportion of adequate antibody concentrations or titers between HIV-infected and HIV-exposed uninfected children (Chi square test or Fisher exact test, as appropriate)

† *P*2: P value for the comparison of the GMC between HIV-infected and HIV-exposed uninfected children (Mann-Whitney test)

‡ GMC = geometric mean concentration and CI = confidence interval, expressed in mUI/ml, except for poliovirus (UI/ml) and pertussis (GMT).

**Table 2:** Low antibody concentrations or titers to the EPI antigens in relation to the percentage of CD4^+^ T cells.

HIV status % of CD4^+^ T cells	Measles			Diphtheria			Tetanus			Pertussis			Poliovirus serotype 3		
	OR*	95% CI†	P	OR	95% CI	*P*	OR	95% CI	*P*	OR	95% CI	*P*	OR	95% CI	*P*
HIV-exposed uninfected (Reference)	1			1			1			1			1		
HIV infected ≥ 25% CD4^+^ T-cells	2.3	0.8–6.5	0.10	2.6	0.9–7.6	0.07	6.7	1.0–43.3	0.045	1.5	0.6–4.0	0.42	13,5	3.1–58.8	0.001
HIV infected <25% CD4^+^ T-cells	11.0	3.1–39.3	<10^−5^	4.1	1.7–10.1	0.002	7.3	1.3–39.9	0.02	4.9	1.8–13.4	0.002	8.7	2.1–35.5	0.003

* Odds ratio

† Confidence interval

**Table 3:** Association between measles antibody concentration and children's characteristics.

Characteristics	Measles antibody		OR*	95% CI†	*P* ‡
	Low (N = 73) n (%)	Adequate (N = 53) n (%)			
Site of inclusion					
Yaoundé	40 (59.7)	27 (40.3)	1.2	0.6–2.4	0.67
Bangui	33 (55.9)	26 (44.1)	1		
HIV status					
Infected	39 (79.6)	10 (20.4)	4.9	2.0–12.0	0.0001
HIV-exposed uninfected	34 (44.2)	43 (55.8)	1		
HIV status–Antiviral therapy (ART)					0.0001
Duration ART <6 months	25 (83.3)	5 (16.7)	6.3	2.0–19.7	0.0003
Duration ART ≥ 6 months	14 (73.7)	5 (26.3)	3.5	1.1–11.2	0.02
HIV-exposed uninfected	34 (44.2)	43 (55.8)	1		
HIV status–CD4^+^ T cells					0.0001
<25% CD4^+^ T-cells	26 (89.7)	3 (10.3)	11.0	3.1–39.3	<10^−5^
≥ 25% CD4^+^ T-cells	13 (65.0)	7 (35.0)	2.3	0.8–6.5	0.10
HIV-exposed uninfected	34 (44.2)	43 (55.8)	1		
HIV status–HIV Viral load					0.0001
Viral load ≥10,000 copies	17 (85.0)	3 (15.0)	7.2	1.8–28.8	0.001
Viral load <10,000 copies	22 (75.9)	7 (24.1)	4.0	1.4–10.9	0.004
HIV-exposed uninfected	34 (44.2)	43 (55.8)	1		
Sex					
Female	43 (56.6)	33 (43.4)	0.9	0.4–1.8	0.70
Male	30 (60.0)	20 (40.0)	1		
Age of children at time of inclusion					
>23 months§	40 (62.5)	24 (37.5)	1.5	0.7–2.0	0.29
≤23 months	33 (53.2)	29 (46.8)	1		
Age at vaccination against measles					
>12 months	10 (71.4)	4 (28.6)	1.9	0.6–6.6	0.28
≤12 months	63 (56.3)	49 (43.7)	1		
Breastfeeding					
Yes	48 (64.9)	26 (35.1)	2.0	1.0–4.2	0.06
No	25 (48.1)	27 (51.9)	1		
Clinical signs					
Yes	18 (72.0)	7 (28.0)	2.2	0.8–5.7	0.11
No	55 (54.5)	46 (45.5)	1		
MUAC|| for age					
<-2Z score (malnutrition)	18 (72.0)	7 (28.0)	2.2	0.8–5.7	0.11
≥-2Z score	55 (54.5)	46 (45.5)	1		
Time since last vaccination					
≥12 months	43 (64.2)	24 (35.8)	1.7	0.8–3.5	0.13
<12 months	30 (50.8)	29 (49.2)	1		
Number of doses received					
2 doses	4 (44.4)	5 (55.6)	0.6	0.1–2.2	0.49
1 dose	69 (59.0)	48 (41.0)	1		
Macrocytosis					
MGV ¶ ≥83 fl	22 (78.6)	6 (21.4)	3.3	1.2–9.1	0.01
MGV¶ <83 fl	50 (52.6)	45 (47.4)	1		
Protidemia					
>85 g/l	17 (77.3)	5 (22.7)	2.8	1.0–8.5	0.05
≤85 g/l	55 (54.5)	46 (45.5)	1		

* Odds ratio of low measles antibody response

† Confidence interval

‡ Chi square test, Fisher exact test or chi square test for trend as appropriate

§ Median age

|| Mid upper arm circumference

¶ Mean globular volume

Multivariate logistic regression models were performed for each EPI vaccine. Only the HIV status-CD4^+^ T-cell percentage was considered in these analysis, due to the high agreement between the composite categorical variables (HIV status-CD4^+^ T-cell percentage and HIV status-VL, k = 0.71 ; HIV status-CD4^+^ T-cell percentage and HIV status-ART, k = 0.75 ; HIV status-VL and HIV status-ART, k = 0.71). For each EPI vaccine, HIV-infected children with severe immunodeficiency (CD4^+^ T-cell percentage <25%) had a significantly higher risk than HIV-exposed uninfected children of having low antibody levels (Table 4). For the poliovirus type 3 vaccine, HIV-infection, whatever the degree of immunodeficiency, was significantly associated with a low antibody titers.

**Table 4:** Logistic regression analysis of various characteristics of children in relation to low antibody concentrations or titers to EPI vaccines (HIV-exposed uninfected children as reference group).

Vaccines/Characteristics of children	*Initial model*			*Final model*		
	OR†	CI‡ 95%	p	OR	CI 95%	p
**Measles (N = 120)**						
HIV status–<25% CD4^+^ T-cells	6.2	(1.5–29.2)	0.01	10.1	(2.8–36.4)	0.0001
HIV status–≥25% CD4^+^ T-cells	2.1	(0.6–7.1)	0.21	2.6	(0.9–7.7)	0.08
Breastfeeding	1.3	(0.6–3.4)	0.44			
Age at vaccination against measles (>12 months)	1.6	(0.4–6.9)	0.51			
Presence of clinical signs	0.8	(0.3–2.8)	0.79			
Malnutrition (MUAC <-2Z scores)	1.2	(0.4–3.8)	0.76			
Time since last vaccination (>12 months)	1.3	(0.6–3.2)	0.52			
Macrocytosis (MGV >85 fl)	2.2	(0.6–7.1)	0.60			
Hyperprotinaemia	1.5	(0.4–5.0)	0.24			
**Diphtheria (N = 126)**						
HIV status–<25% CD4^+^ T-cells	3.4	(1.2–10.0)	0.03	3.8	(1.5–9.5)	0.004
HIV status–≥25% CD4^+^ T-cells	1.6	(0.5–5.7)	0.44	2.4	(0.8–6.9)	0.12
Inclusion site (Yaoundé)	2.2	(0.8–5.6)	0.11			
Sex (female)	1.7	(0.7–4.1)	0.21			
Malnutrition (MUAC <-2Z scores)	1.4	(0.5–4.4)	0.52			
Age (>23 months)	1.1	(0.3–4.2)	0.91			
Time since last vaccination (≥18 months)	1.7	(0.4–6.7)	0.44	2.3	(1.0–5.1)	0.04
Number of doses received (<4 doses)	2.4	(0.5–11.2)	0.27			
Macrocytosis (MGV >85 fl)	1.0	(0.4–3.0)	0.94			
**Pertussis (N = 125)**						
HIV status–<25% CD4^+^ T-cells	2.6	(0.8–9.2)	0.12	3.8	(1.3–11.6)	0.02
HIV status–≥25% CD4^+^ T-cells	0.9	(0.2–3.0)	0.81	1.3	(0.4–3.7)	0.63
Inclusion site (Yaoundé)	1.5	(0.6–3.8)	0.35			
Sex (female)	1.0	(0.4–2.3)	0.97			
Malnutrition (MUAC <-2Z scores)	1.1	(0.4–3.6)	0.83			
Presence of clinical signs	6.1	(1.5–24.7)	0.01	4.8	(1.3–17.6)	0.02
Number of doses received (<4 doses)	6.0	(1.5–23.7)	0.01	6.8	(1.8–25.4)	0.004
Time since last vaccination (≥18 months)	1.4	(0.5–4.7)	0.40			
Macrocytosis (MGV >85 fl)	1.5	(0.5–4.7)	0.44			
**Tetanus (N = 126)**						
HIV status–<25% CD4^+^ T-cells	6.6	(0.6–55.6)	0.12	6.9	(1.1–42.0)	0.04
HIV status–≥25% CD4^+^ T-cells	2.5	(0.3–17.4)	0.46	4.3	(0.6–30.9)	0.14
Inclusion site (Yaoundé)	8.1	(0.6–106.1)	0.10			
Sex (female)	8.6	(0.8–86.6)	0.07	8.5	(1.0–75.2)	0.05
Age (>23 months)	12.1	(0.6–257.9)	0.11	8.4	(1.0–72.3)	0.05
Malnutrition (MUAC <-2Z scores)	4.0	(0.4–34.7)	0.22			
Breastfeeding (yes)	0.4	(0.05–2.7)	0.33			
Time since last vaccination (≥18 months)	0.6	(0.04–8.0)	0.69			
Macrocytosis (MGV >85 fl)	0.7	(0.1–4.3)	0.72			
**Polio serotype 3 (N = 123)**						
HIV status–<25% CD4^+^ T-cells	3.8	(0.7–22.6)	0.14	7.2	(1.7–30.2)	0.007
HIV status–≥25% CD4^+^ T-cells	9.4	(1.6–54.0)	0.01	13.8	(3.1–60.9)	0.001
Inclusion site (Yaoundé)	0.9	(0.2–3.7)	0.86			
Sex (female)	1.1	(0.3–3.7)	0.91			
Malnutrition (MUAC <-2Z scores)	1.3	(0.3–5.2)	0.74			
Presence of clinical signs	1.6	(0.4–7.5)	0.52			
Time since last vaccination (≥18 months)	0.7	(0.2–2.7)	0.64			
Macrocytosis (MGV >85 fl)	1.7	(0.5–6.3)	0.41			
Hyperprotinaemia	1.4	(0.4–5.3)	0.62			

† Adjusted Odds Ratio

‡ Confidence interval

## Discussion

Soon, the introduction of ART for infants and children living in resource-constrained settings should considerably prolong their life expectancy and reduce HIV disease progression. The question of the long-lasting antibody persistence to EPI vaccines in this population should now be addressed. In this study, we evaluated the persistence of antibody levels following routine EPI vaccine administration in perinatally HIV-infected and HIV-exposed uninfected children in Central Africa. We found that HIV-infected children had significantly lower levels of antibodies to the EPI vaccines than HIV-exposed uninfected children. On one hand, the proportion of HIV-infected children with expected antibody concentrations to measles was substantially low; although HIV-infected responders had GMC similar to the HIV-exposed uninfected children. On the other hand, although similar proportions of HIV-infected and HIV-exposed uninfected children were found adequate to the poliovirus types 1 and 2 vaccines, the antibody GMT were lower in HIV-infected children.

In this study, half of the children (44/88) in CAR could not be included because they did not meet the criteria about the EPI vaccine schedule showing how much a successful EPI could be difficult to implement and to maintain at a national level. Logistics related to supply, storage and administration of vaccines can lead to difficulty in applying the EPI recommended schedule. A recent study in Zambia showed a lower rate of EPI vaccine coverage in HIV-infected children [13]. Moreover, EPI vaccines are provided free-of-charge only until 12 months of age and we showed that, consequently, booster doses were almost never administrated in routine practice as they must be paid for by the families. Nevertheless, we observed that the few HIV-infected children who received four doses of the DTwP vaccine had a better antibody concentration than those who received only the primary series of three doses. For the measles vaccine, in sub-Saharan African countries, the first dose of the measles vaccine is recommended at 9 months of age to increase the protection of younger children and a second dose, known to improve the level of immunization, should be administered. Supplemental immunization activities (SIA) have been successful in controlling measles in southern Africa despite a high HIV prevalence [14], [15]; but there were no SIA and the wild-type measles virus still circulating in the two countries at the time of our study.

Low responsiveness to the measles vaccine in HIV-infected children has been reported previously [6], [16]–[18]. Attempts to use a high-dose Edmonston-Zagreb measles vaccine showed a higher conversion rate [19]; however, the introduction of high-dose vaccines was abandoned because of safety risks [20]. Waibale *et al*. linked low antibody response to the measles vaccine to nutritional status (stunting) rather than to HIV infection [21]. We did not include children with very poor general health status in our study to avoid collecting blood of children with short life-time expectancy or with on-going infectious disease, which may explain why we did not observe this link with the nutritional status. In routine vaccination practice, we observed a low persistence of antibody to the measles vaccine in all children; this was particularly marked in HIV-infected children. However, in terms of clinical protection, one cannot predict if HIV-infected children with no antibodies to measles despite immunization remain susceptible or not to measles infection. A recent study demonstrated a very high mortality level due to measles epidemics in unvaccinated children from Niger, Nigeria and Chad with case fatality rates of 4–10% in children under 5 years old [22], thus reminding the devastating impact of measles in unvaccinated populations [23]. In HIV-uninfected children born to HIV-uninfected mothers, the cellular response to the measles vaccine appears to be induced even after early immunization (at six months of age), whereas the antibody response remains low or not detectable at this age [24], [25]. Once established, T-cell memory against measles is long lasting in HIV-uninfected individuals [26]. However, the CD4^+^ T-cell deficiency in HIV-infected children likely impairs mounting an effective memory against vaccines, and secondary vaccine failure due to a decline in vaccine-induced antibody over time has been described [27]–[29].

The poor antibody levels to EPI vaccines observed in perinatally HIV-infected children is probably related to major immunological dysfunction involving both cellular and humoral responses. The peripartum transmission of HIV correlates with rapid disease progression and a high mortality rate in HIV-infected infants [30]. In adults, it has been recently shown that acute HIV infection results in a massive and irreversible depletion of mucosal CD4^+^ memory T cells, whereas the regenerative capacity of resting central memory and naïve T cells remains largely intact [reviewed in 31], [32]. Systemic immune activation is impaired after such a profound depletion of CD4^+^ memory T cells in the mucosa, affecting pathogen-specific adaptive responsiveness. These mechanisms have not been studied in HIV-infected children born to HIV-infected mothers. Children were examined at a time when their antibody concentrations would have peaked and still not undergone significant decay, suggesting either a lack of inducing immediate vaccine response or, at least, a lack of immune capacity to maintain an adequate antibody level.

We included, as a reference group, perinatally HIV-exposed uninfected children; this was initially for practical reason because the HIV status of the mothers and the children were already determined at the time of the cross-sectional study. We expected similar antibody concentrations in this reference group and in the HIV unexposed and uninfected children [33], [34]. However, a recent study demonstrates the influence of the maternal HIV status on the occurrence of severe pneumonia in young South African children [35]. Little is known about immune activation and lymphocyte homeostasis in HIV-uninfected children exposed to HIV during pregnancy. It was previously shown that cell-mediated immunity and T-cell maturation are altered in HIV-uninfected infants born to HIV-infected women [36]. More recently, immune activation and skewing of post-thymic differentiation were evidenced in healthy adolescents born to HIV-infected mothers [37]. This might explain, in part, the low antibody concentrations to the measles vaccine (around 60% of adequate response) observed in HIV-exposed uninfected children.

We also observed that the persistence of antibody to the TOPV, particularly to types 1 and 2 was less affected in HIV-infected children than to the other EPI vaccines, as reported previously [38], [39]. The influence of the degree of immunodeficiency has not been demonstrated for the TOPV. This may be due to the administration of multiple TOPV doses during yearly national poliomyelitis immunization campaigns performed in Cameroon and in CAR for children under 5 years of age. During these campaigns, children received two drops of the TOPV whatever their routine vaccination status. However, although the proportion of children with antibodies to polioviruses types 1 and 2 were high in both HIV-infected and HIV-exposed uninfected children, the antibody titers were clearly lower in HIV-infected children even after supplemental doses of the TOPV. On the contrary, the proportion of HIV-infected children with antibodies to poliovirus type 3 was lower as compared to serotypes 1 and 2, but the titers were similar in both children groups. Lower antibody titers to poliovirus type 3 as compared to types 1 and 2 have been reported previously in HIV-uninfected children [40], [41].

We unambiguously observed that the proportion of children with low antibodies levels to the EPI vaccines was associated with the severity of HIV-induced immunosuppression. The benefits of using ART for HIV-infected children [42] and of introducing ART to infants at an early stage [43], [44], in terms of morbidity and mortality, have been now documented. In young children receiving ART, we also anticipate the improvement of the immune response to the EPI vaccines. A prospective study combining early ART therapy and the administration of an EPI vaccine schedule completed by a supplemental measles vaccine dose and reinforcing the schedule by booster doses of DTwP and TOPV vaccines is needed. Aside the EPI vaccines, responses to *H. influenzae* type b conjugate, hepatitis B, pneumococcus and yellow fever vaccines also deserve further investigation in this population. Our findings also highlight the urgent need to delineate the mechanisms of cellular and humoral responses to EPI vaccines in HIV-infected children.

## References

[1] WHO/UNAIDS. Report on the global AIDS epidemic (2006). http://www.unaids.org/en/HIV_data/2006GlobalReport.

[2] WHO Immunization service delivery and accelerated disease control. Vaccine Preventable Diseases.. http://www.who.int/immunization_delivery/vaccinesviatheInternet.

[3] Moss WJ, Clements CJ, Halsey NA (2003). Immunization of children at risk of infection with human immunodeficiency virus.. Bull World Health Organ.

[4] Pancharoen C, Ananworanich J, Thisyakorn U (2004). Immunization for persons infected with human immunodeficiency virus.. Curr HIV Res.

[5] Rouet F, Ekouevi DK, Chaix ML, Burgard M, Inwoley A (2005). Transfer and evaluation of an automated, low-cost real-time reverse transcription-PCR test for diagnosis and monitoring of human immunodeficiency virus type 1 infection in a West African resource-limited setting.. J Clin Microbiol.

[6] Scott S, Cumberland P, Shulman CE, Cousens S, Cohen BJ (2005). Neonatal measles immunity in rural Kenya: the influence of HIV and placental malaria infections on placental transfer of antibodies and levels of antibody in maternal and cord serum samples.. J Infect Dis.

[7] Cohen BJ, Parry RP, Doblas D, Samuel D, Warrener L (2006). Measles immunity testing: comparison of two measles IgG ELISAs with plaque reduction neutralisation assay.. J Virol Methods.

[8] Relyveld E, Oato NH, Guerin N, Coursaget P, Huet M (1991). Determination of circulating antibodies directed to pertussis toxin and of agglutinogens in children vaccinated with either the whole cell or component pertussis vaccine in France, Japan and Senegal.. Vaccine.

[9] WHO (1997). Neutralisation test for polio antibodies. Manual for the virological investigation of poliovirus (Chapter 6).

[10] WHO http://www.who.int/hiv/pub/guidelines/WHOpaediatric.pdf.

[11] Dunn D, HIV Paediatric Prognostic Markers Collaborative Study Group (2003). Short-term risk of disease progression in HIV-1-infected children receiving no antiretroviral therapy or zidovudine monotherapy: a meta-analysis.. Lancet.

[12] de Onis M, Yip R, Mei Z (1997). The development of MUAC-for-age reference data recommended by a WHO Expert Committee.. Bull World Health Organ.

[13] Setse RW, Cutts F, Monze M, Ryon JJ, Quinn TC (2006). HIV-1 Infection as a risk factor for incomplete childhood immunization in Zambia.. J Trop Pediatr.

[14] Helfand RF, Moss WJ, Harpaz R, Scott S, Cutts F (2005). Evaluating the impact of the HIV pandemic on measles control and elimination.. Bull World Health Organ.

[15] Wolfson LJ, Strebel PM, Gacic-Dobo M, Hoekstra EJ, McFarland JW (2007). Has the 2005 measles mortality reduction goal been achieved? A natural history modelling study.. Lancet.

[16] Rudy BJ, Rutstein RM, Pinto-Martin J (1994). Responses to measles immunization in children infected with human immunodeficiency virus.. J Pediatr.

[17] Arpadi SM, Markowitz LE, Baughman AL, Shah K, Adam H (1996). Measles antibody in vaccinated human immunodeficiency virus type 1-infected children.. Pediatrics.

[18] Lindgren-Alves CR, Freire LM, Oliveira RC, Guerra HL, Da-Silva EE (2001). Search of antimeasles antibodies in HIV-infected children after basic immunization.. J Pediatr (Rio J).

[19] Lepage P, Dabis F, Msellati P, Hitimana DG, Stevens AM (1992). Safety and immunogenicity of high-dose Edmonston-Zagreb measles vaccine in children with HIV-1 infection. A cohort study in Kigali, Rwanda.. Am J Dis Child.

[20] Garenne M, Leroy O, Beau JP, Sene I (1991). Child mortality after high-titre measles vaccines: prospective study in Senegal.. Lancet.

[21] Waibale P, Bowlin SJ, Mortimer EA, Whalen C (1999). The effect of human immunodeficiency virus-1 infection and stunting on measles immunoglobulin-G levels in children vaccinated against measles in Uganda.. Int J Epidemiol.

[22] Grais RF, Dubray C, Gerstl S, Guthmann JP, Djibo A (2007). Unacceptably high mortality related to measles epidemics in Niger, Nigeria, and Chad.. PLoS Med.

[23] Moss WJ (2007). Measles still has a devastating impact in unvaccinated populations.. PLoS Med 2007.

[24] Bautista-Lopez N, Ward BJ, Mills E, McCormick D, Martel N (2000). Development and durability of measles antigen-specific lymphoproliferative response after MMR vaccination.. Vaccine.

[25] Gans H, Yasukawa L, Rinki M, DeHovitz R, Forghani B (2001). Immune responses to measles and mumps vaccination of infants at 6, 9, and 12 months.. J Infect Dis.

[26] Naniche D, Garenne M, Rae C, Manchester M, Buchta R (2004). Decrease in measles virus-specific CD4 T cell memory in vaccinated subjects.. J Infect Dis.

[27] Al-Attar I, Reisman J, Muehlmann M, McIntosh K (1995). Decline of measles antibody titers after immunization in human immunodeficiency virus-infected children.. Pediatr Infect Dis J.

[28] Brunell PA, Vimal V, Sandu M, Courville TM, Daar E (1995). Abnormalities of measles antibody response in human immunodeficiency virus type 1 (HIV-1) infection.. J Acquir Immune Defic Syndr Hum Retrovirol..

[29] Bekker V, Scherpbier H, Pajkrt D, Jurriaans S, Zaaijer H (2006). Persistent humoral immune defect in highly active antiretroviral therapy-treated children with HIV-1 infection: loss of specific antibodies against attenuated vaccine strains and natural viral infection.. Pediatrics.

[30] Newell ML, Coovadia H, Cortina-Borja M, Rollins N, Gaillard P (2004). Mortality of infected and uninfected infants born to HIV-infected mothers in Africa: a pooled analysis.. Lancet.

[31] Grossman Z, Meier-Schellersheim M, Paul WE, Picker LJ (2006). Pathogenesis of HIV infection: what the virus spares is as important as what it destroys.. Nat Med.

[32] Brenchley JM, Price DA, Douek DC (2006). HIV disease: fallout from a mucosal catastrophe?. Nat Immunol.

[33] Lepage P, Msellati P, Hitimana DG, Bazubagira A, Van Goethem C (1996). Growth of human immunodeficiency type 1-infected and uninfected children: a prospective cohort study in Kigali, Rwanda, 1988 to 1993.. Pediatr Infect Dis J.

[34] Taha TE, Graham SM, Kumwenda NI, Broadhead RL, Hoover DR (2000). Morbidity among human immunodeficiency virus-1-infected and -uninfected African children.. Pediatrics.

[35] McNally LM, Jeena PM, Gajee K, Thula SA, Sturm AW (2007). Effect of age, polymicrobial disease, and maternal HIV status on treatment response and cause of severe pneumonia in South African children: a prospective descriptive study.. Lancet.

[36] Clerici M, Saresella M, Colombo F, Fossati S, Sala N (2000). T-lymphocyte maturation abnormalities in uninfected newborns and children with vertical exposure to HIV.. Blood.

[37] Vigano A, Saresella M, Schenal M, Erba P, Piacentini L (2007). Immune activation and normal levels of endogenous antivirals are seen in healthy adolescents born of HIV-infected mothers.. AIDS.

[38] Barbi M, Biffi MR, Binda S, Clerici-Schoeller M, Ferraris G (1992). Immunization in children with HIV seropositivity at birth: antibody response to polio vaccine and tetanus toxoid.. AIDS.

[39] Ryder RW, Oxtoby MJ, Mvula M, Batter V, Baende E (1993). Safety and immunogenicity of bacille Calmette-Guerin, diphtheria-tetanus-pertussis, and oral polio vaccines in newborn children in Zaire infected with human immunodeficiency virus type 1.. J Pediatr.

[40] Sutter RW, Suleiman AJ, Malankar P, Al-Khusaiby S, Mehta F (2000). Trial of a supplemental dose of four poliovirus vaccines.. N Engl J Med.

[41] Triki H, Abdallah MV, Ben Aissa R, Bouratbine A, Ben Ali Kacem M (1997). Influence of host related factors on the antibody response to trivalent oral polio vaccine in Tunisian infants.. Vaccine.

[42] Fassinou P, Elenga N, Rouet F, Laguide R, Kouakoussui KA (2004). Highly active antiretroviral therapies among HIV-1-infected children in Abidjan, Cote d'Ivoire.. AIDS.

[43] Faye A, Le Chenadec J, Dollfus C, Thuret I, Douard D (2004). Early versus deferred antiretroviral multidrug therapy in infants infected with HIV type 1.. Clin Infect Dis.

[44] Chiappini E, Galli L, Tovo PA, Gabiano C, Gattinara GC (2006). Virologic, immunologic, and clinical benefits from early combined antiretroviral therapy in infants with perinatal HIV-1 infection.. AIDS.

